# Characterization of an imported HIV-1 A1/A7/G recombinant in China

**DOI:** 10.1186/s12985-023-02274-x

**Published:** 2024-01-04

**Authors:** Qing-Hai Li, Yun-Qi Zhang, En-Long Li, Qi Guo, Xiao-Hong Chen, Fu-Xiang Wang, Jia-Ye Wang

**Affiliations:** 1 Genomics Research Center, College of Pharmacy, Harbin Medical University, Harbin, China; 2 Department of Microbiology, Harbin Medical University, 157 Baojian Road, 150001 Harbin, China; 3 Heilongjiang Provincial Key Laboratory of Infection and Immunity, Harbin, China; 4 Department of Infectious Diseases, the Fourth Affiliated Hospital of Harbin Medical University, Harbin, China; 5 Department of Infectious Diseases, the Second Affiliated Hospital of Southern University of Science and Technology, Shenzhen, China; 6 Heilongjiang Academy of Medical Sciences, Harbin, China

**Keywords:** HIV-1, Sub-subtype A1, Sub-subtype A7, Subtype G, Recombinant

## Abstract

**Background:**

International migration has accelerated the HIV-1 spread across national borders, gradually reducing the restrictions on the geographical distribution of HIV-1 subtypes. Subtypes A and G are globally recognized as the third and sixth most dominant HIV-1 genotypes, mainly prevalent in Africa, but rarely detected in China. Here we reported an imported HIV-1 recombinant which was composed of sub-subtypes A1 and A7 of subtype A and subtype G genes in a Chinese female. This virus was the first HIV-1 recombinant including A7 genes reported in the world.

**Case presentation:**

The near full-length genome (NFLG) was obtained from the plasma sample of the female in an HIV-1 molecular epidemiological survey with 853 participants in China. Phylogenetic analyses showed that this NFLG sequence contains three A7 segments, four G segments and one A1 segment with seven breakpoints, and all these segments were closely related to HIV-1 references circulating in Africa. The evidence from epidemiological investigation indicated that this female participant had a more-than-two-years heterosexual contact history with a fixed partner from Nigeria, a country in west Africa, which further supported the results of phylogenetic analyses. By the Bayesian phylogenetic analyses, the times of most recent common ancestors (tMRCA) of the partial *pol* gene (nt2308-3284, A7 region) and full-length *vpr*-*vpu* plus partial *env* gene (nt5534-6858, G region) were estimated around 1989 and 1984, respectively.

**Conclusions:**

In this study, by using the NFLG sequencing, we identified an imported HIV-1 A1/A7/G recombinant which was estimated to originate around 1980s in Africa and introduced into China with international migration. This study highlighted the complexity of the global HIV-1 epidemic, the necessity of using genome sequences to determine HIV-1 genotypes and the importance of real-time monitoring of HIV-1 infection among international migrants and travelers.

## Background

The human immunodeficiency virus type 1 (HIV-1) M group was classified into 10 subtypes (A-D, F-H, J, K and L) and several sub-subtypes according to the genetic clustering patterns of HIV-1 sequences. Inter-subtype recombination of HIV-1 plays a significant role in the genetic diversity of the virus, exerting a profound impact on the virus’s infectivity, pathogenicity and drug susceptibility [1]. Identifying recombinant strains of HIV-1 at the full-length genome level contributes to a deeper understanding of the viral evolution and transmission dynamics, thereby providing valuable insights for the development of prevention and control strategies.

According to a systematic review and global survey with 383,519 samples from 116 countries, subtypes A and G have become the third and sixth most dominant HIV-1 subtypes, responsible for 10.3% and 4.6% of global infections, respectively, during 2010–2015 [2, 3]. Subtype A is now further divided into seven sub-subtypes (A1-A4 and A6-A8) [4]. Among them, A1 is widely distributed around the world, accounting for approximately 70% of global subtype A infections, and A7 was initially proposed as a separate sub-subtype of subtype A in 2018 [5]. Up to August 8, 2023, a total of 51 A7 sequences (including 4 near full-length genomes (NFLGs), 2 full-length *gag* genes and 45 partial *pol* genes) have been documented in the HIV database, and most of them (37/51, 72.5%) were identified in Nigeria, a country located in west Africa [4].

Subtypes A and G were rarely detected in China. Subtype A was firstly reported among individuals who had returned after working in Africa in 1997 [6], and subtype G was firstly identified in a Chinese woman who had a foreign sexual partner in 2000 [7]. According to the results of the third national epidemiological survey in 2006, subtypes A and G were responsible for 0.06% and 0.03% of HIV-1 infections in China [8]. So far, among the sub-subtypes of subtype A, A1, A2, A3 and A6 have been recorded in China, but A4, A7 and A8 have not [9].

Our team conducted HIV-1 molecular epidemiological surveillance using partial *pol* genes (HXB2: nt2147-3462) derived from antiretroviral therapy-naïve individuals who were newly diagnosed as HIV-1 infection during 2017–2019 in Heilongjiang province of China. Among a total of 853 samples, we found one *pol* gene which might be identified as A7. After the revelation of its NFLGs, it was ascertained that this represents, in actuality, an imported unique recombinant form (URF) of A1/A7/G in China. Notably, the presence of an A7 gene had not been previously reported in China, and no such HIV-1 recombinants had been identified worldwide. This study aimed to elucidate the genomic composition of this virus and trace its origin.

## Case presentation

The virus was isolated from a 24-year-old female who was firstly diagnosed as HIV-1 sero-positive in 2017 at the Fourth Affiliated Hospital of Harbin Medical University. She self-reported that she acquired HIV-1 by heterosexual contact and had no histories of intravenous drug use, blood transfusion or other high-risk behaviors. The peripheral whole blood of this participant was then collected for subsequent analysis before the initiation of antiretroviral therapy.

From the plasma sample of this participant, a NFLG (sequence ID: HLJ17137) was isolated as previously described [10, 11]. In brief, Viral RNA was extracted from 140 µl of the plasma and was used to synthesize cDNA by reverse transcription reaction according to the instruction of the PrimeScript™ II First Strand cDNA Synthesis Kit (TaKaRa Bio, Inc., Beijing, China). Then, using the cDNA as the template, the NFLG was amplified into two segments with an approximately 500 bp overlapping region by nested PCR with four pairs of primers. The PCR products were purified and subjected to direct sequencing by ABI 3730XL DNA sequencer (Applied Biosystems, Carlsbad, CA) in Suzhou GENEWIZ Company.

This NFLG was 8694 bp in length and positioned nt790-9417 relative to HXB2 genome. By the online Basic Local Alignment Search Tool (BLAST) in HIV database (https://www.hiv.lanl.gov/content/sequence/BASIC_BLAST/basic_blast.html), we found the most similar sequence to HLJ17137 (with a highest identity of 87%), a circulating recombinant form (CRF) 02_AG virus (Genbank accession no. AB485634), which was isolated in 1991, indicating that HLJ17137 might be an A/G recombinant virus. Then HLJ17137 sequence was deposited in Genbank with an accession number OR139611.

**Fig. 1:**
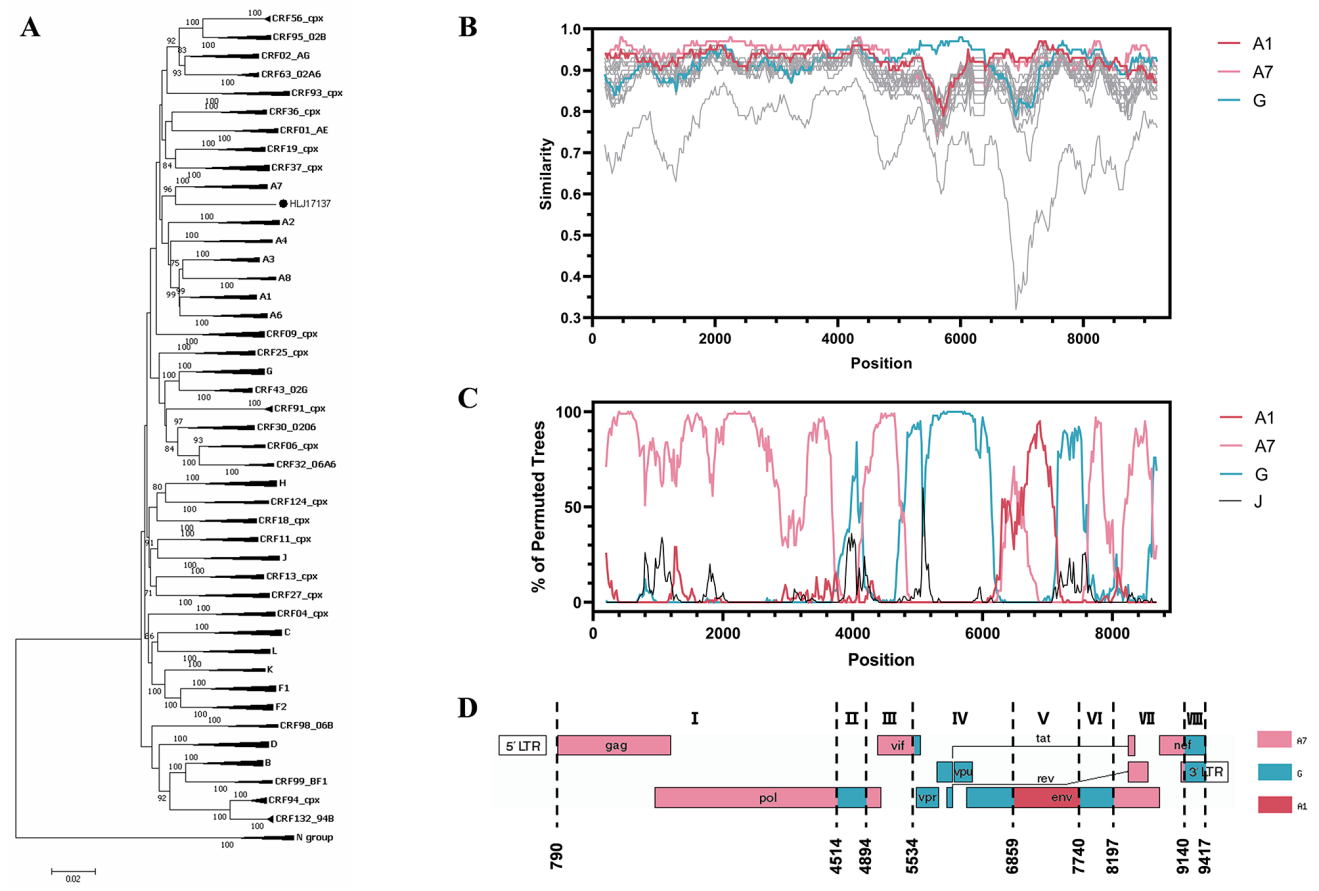
Phylogenetic and recombination analysis of HLJ17137. (**A**) The near full-length genome (NFLG) of HLJ17137 virus was aligned with representative references of HIV-1 subtypes A, G and CRFs containing A and G genes using MAFFT 7 (https://mafft.cbrc.jp/alignment/server/index.html). A phylogenetic tree was then constructed using neighbor-joining method based on Kimura 2-parameter model with 1000 bootstrap replicates in Mega 7.0.26 software. The HLJ17137 sequence is marked with a black dot. (**B**) The similarity plot analysis was carried out by Simplot 3.5.1 software. Sub-subtypes A1, A7 and subtype G were colored according to the legend, while the others (A2-A4, A6, A8, B-D, F1, F2, H, J, K, L and N) were not colored for the convenience of reading. (**C**) Bootscan analysis was performed using a window size of 400 bp and a step size of 20 bp, together with reference sequences of sub-subtypes A1, A7 and subtype G, and an out-group control of subtype J. (**D**) Genomic map of HLJ17137 was visualized with the Recombinant HIV-1 Drawing Tool (https://www.hiv.lanl.gov/content/sequence/DRAW_CRF/recom_mapper.html).

Phylogenetic analysis of NFLG sequences showed that HLJ17137 was closely related to subtype A, especially A7 (Fig. 1A). The recombination structure analysis using the Simplot 3.5.1 suggested that HLJ17137 has high sequence similarity with A1, A7 and G in different regions of its genome (Fig. 1B). The mosaic structure of HLJ17137 was finally confirmed by Bootscan analysis and displayed as follows: I_A7_ (nt790-4513), II_G_ (nt4514-4893), III_A7_ (nt4894-5533), IV_G_ (nt5534-6858), V_A1_ (nt6859-7739), VI_G_ (nt7740-8196), VII_A7_ (nt8197-9139), VIII_G_ (nt9140-9417). The positions of breakpoints were numbered according to HXB2 genome (Fig. 1C and D).

**Fig. 2:**
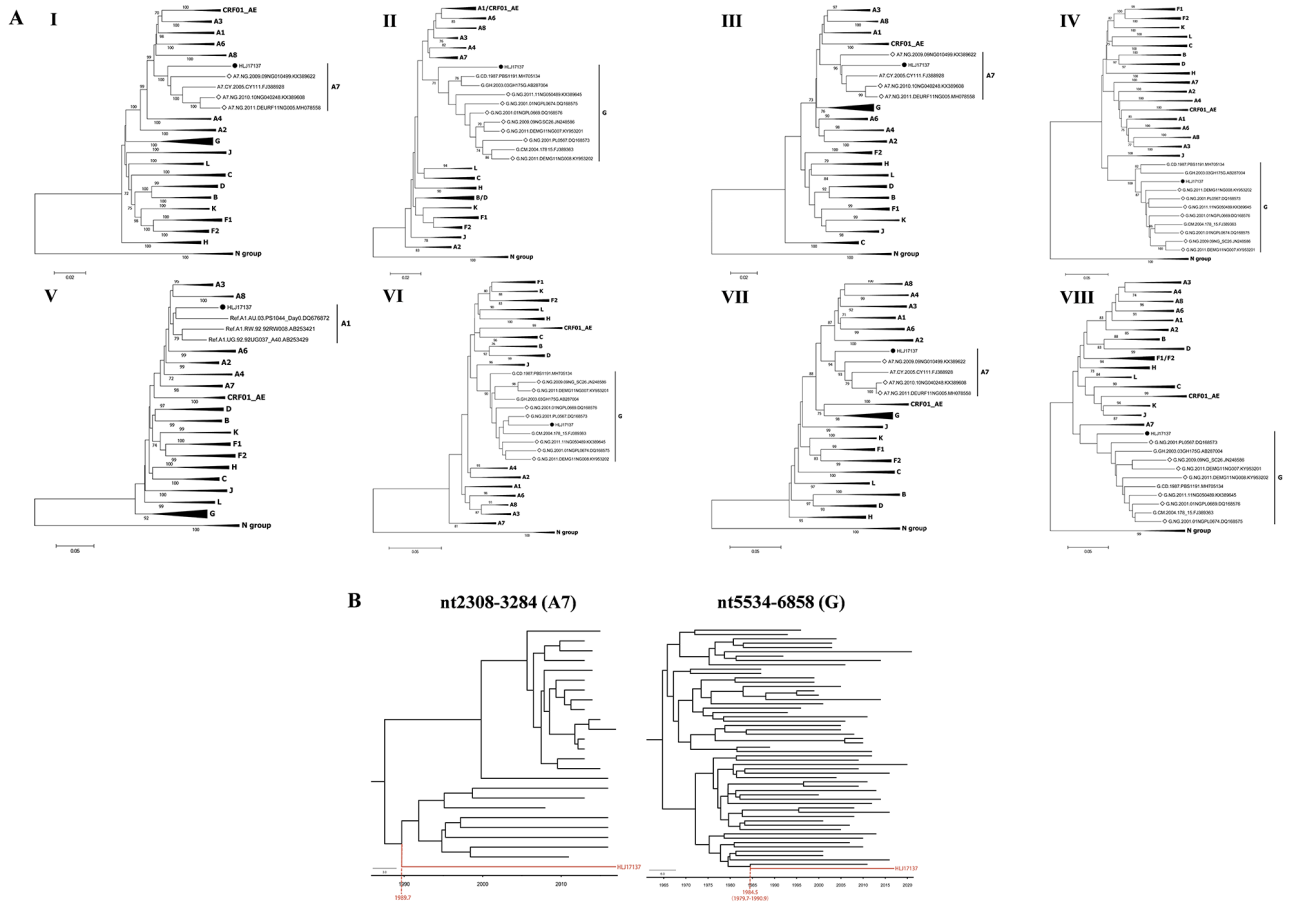
Subregion phylogenetic analyses and maximum clade credibility (MCC) trees of HLJ17137. (**A**) The neighbor-joining (NJ) trees of eight fragments (I-VIII) of HLJ17137 were constructed based on Kimura 2-parameter model with 1000 bootstrap replicates in Mega 7.0.26 software. The HLJ17137 sequence is marked with a black dot. The reference strains sampled from Nigeria are marked with hollow diamonds. (**B**) ​MCC trees for the regions corresponding to the HXB2 genome positions nt2308-3284 and nt5534-6858. The MCC trees were constructed by Beast v18.4. Timescale is shown at the bottom of the tree. The tMRCA and 95% highest probability density (HPD) for the key nodes were displayed. HLJ17137 sequence is highlighted in red.

To verify the origin of this recombinant virus, further phylogenetic analyses were performed for the subregions of HLJ17137, which revealed that A7 segments (I, III and VII) and G segments (II, IV, VI and VIII) were closely related to references circulating in Nigeria, Ghana and Cameroon in West Africa, and the short A1 segment (V) was most closely related to references from East Africa (Rwanda and Uganda) or Australia (Fig. 2A). These results implied that the HLJ17137 might be an imported virus from Africa, rather than a local strain circulating in Heilongjiang, China.

To estimate the time of emergence of HLJ17137 virus, Bayesian phylogenetic analyses under the uncorrelated log-normal relaxed clock with the GTR + G + I model and a constant size coalescent tree prior were then carried out with two regions: nt2308-3284 (A7 segment) and nt5534-6858 (G segment). The Markov chain Monte Carlo (MCMC) methods were executed for 10 million steps with sampling every 1000 steps. The effective sample size (ESS) of each parameter was > 200.The initial 10% of the trees of MCMC analysis were discarded as burn in. The Maximum Clade Credibility (MCC) tree was summarized by Tree Annotator v1.10.4 and shown by FigTree v1.4.4. As shown in Fig. 2B, the times of most recent common ancestors (tMRCA) of two segments were inferred to be around 1989.7 and 1984.5, respectively, revealing that HLJ17137 likely originated around the 1980s.

An in-depth epidemiological investigation of the participant was also conducted. The participant claimed that she lived mainly in Heilongjiang, China and had never been abroad. During February 2014 to April 2016, she had a fixed sexual partner from Nigeria without knowing his status of HIV-1 infection. After April 2016, she self-reported having only one sexual partner and they had never tested for HIV-1. In the following tracking, we failed to confirm the HIV-1 status of her former partner because he had returned to Nigeria in April 2016, and her current sexual partner tested negative for HIV-1 antibody.

## Discussion and conclusions

Globalization and international migration have facilitated the HIV-1 spread across national borders, gradually reducing the restrictions on the geographical distribution of HIV-1 subtypes [12]. China is one of the countries with a high diversity of HIV-1 subtypes in the world. The CRF07_BC, previously confined to China, has spread to other Asian countries and outside of Asia [13]. And, several unique HIV-1 strains, including sub-subtype A6 and CRF14_BG from Europe, CRF06_cpx from Africa, and CRF12_BF from South America, have been imported into China [14–17]. In the present study, a new imported A1/A7/G virus, HLJ17137, was identified in China. The evidences of phylogenesis and epidemiological analyses indicated that this virus might be from Africa and imported into China by the international migration.

Africa is the region most affected by HIV-1 in the world. The greatest genetic diversity of HIV-1 has been found in west and central Africa, where subtype A (including A1, A7 and other sub-subtypes), G and CRF02_AG co-circulate with many other subtypes and CRFs [2]. Co-circulation of different subtypes/CRFs facilitates the generation of HIV-1 recombinants. Hence, it is not surprising that the recombination of A1, A7, G viruses could emerge in Africa. What really makes us surprised is that either the HLJ17137 virus or other recombinants containing A7 genes were not previously reported in regions where A7 viruses have been found, especially in Nigeria which currently has the highest reported number of A7 viruses. The fact is that most known A7 viruses (92.2%, 47/51) were identified based on short fragments of *pol* gene (HXB2: nt2045-3869) and/or *gag* gene (HXB2: nt790-2292) [4], and both of these two regions were genetically classified as A7 in HLJ17137. The unavailability of full-length genomes makes it difficult to determine the presence or absence of inter-subtype recombination events in these “A7” viruses.

In this study, we described the genetic characterization of the first HIV-1 A1/A7/G recombinant which was estimated to originate around 1980s in Africa and subsequently introduced into China with international migration. This study highlighted the complexity of the HIV-1 epidemic in the world, the necessity of using genome sequences to determine HIV-1 genotypes, particularly when the number of referable sequences is extremely limited, and the importance of real-time monitoring of HIV-1 infection among international migrants and travelers.

## Data Availability

The sequence generated and analyzed during the current study are available in the National Center for Biotechnology Information repository with a Genbank accession number OR139611. (https://www.ncbi.nlm.nih.gov)

## Abbreviations

BLAST: Basic Local Alignment Search Tool
CRF: Circulating recombinant form
HIV-1: Human immunodeficiency virus type 1
NFLG: Near full-length genome
tMRCA: The time of most recent common ancestor
URF: Unique recombinant form
